# Supplementary material for: Exploring lumbar and lower limb kinematics and kinetics for evidence that lifting technique is associated with LBP
Source: PLoS One. 2021 Jul 21;16(7):e0254241. doi: 10.1371/journal.pone.0254241 (PMC8294511; doi:10.1371/journal.pone.0254241)
Supplement: S1 Table — (DOCX) [file pone.0254241.s001.docx]

**S2 Table. Marker locations.**

| **Marker** | **Description** | **Notes** |
| --- | --- | --- |
| LUMBAR MARKERS | | |
| L1 |  |  |
| L3 |  |  |
| L5 |  |  |
| lateral to L2 - Right |  |  |
| lateral to L2 - Left |  |  |
| lateral to L4 - Right |  |  |
| lateral to L4 - Left |  |  |
| PELVIS MARKERS | | |
| LASI | Left anterior superior iliac spine |  |
| RASI | Right anterior superior iliac spine |  |
| LPSI | Left posterior superior iliac spine |  |
| RPSI | Right posterior superior iliac spine |  |
| LIC | Left iliac crest |  |
| RIC | Right iliac crest |  |
| LOWER LIMBS | | |
| Left thigh cluster | LTH1, LTH2, LTH3 |  |
| Right thigh cluster | RTH1, RTH2, RTH3 |  |
| Left tibia cluster | LTB1, LTB2, LTB3, LTB4 |  |
| Right tibia cluster | RTB1, RTB2, RTB3, RTB4 |  |
| LLFC | Left lateral femoral condyle | Virtual marker |
| LMFC | Left medial femoral condyle | Virtual marker |
| RLFC | Right lateral femoral condyle | Virtual marker |
| RMFC | Right medial femoral condyle | Virtual marker |
| LLMAL | Left lateral malleolus | Virtual marker |
| LMMAL | Left medial malleolus | Virtual marker |
| RLMAL | Right lateral malleolus | Virtual marker |
| RMMAL | Right medial malleolus | Virtual marker |
| LCAL | Left calcaneus |  |
| LCAL1 | Left calcaneus 1 | Virtual marker |
| LMT1 | Left 1^st^ metatarsal |  |
| LMT5 | Left 5^th^ metatarsal |  |
| RCAL | Right calcaneus |  |
| RCAL1 | Right calcaneus 1 | Virtual marker |
| RMT1 | Right 1^st^ metatarsal |  |
| RMT5 | Right 5^th^ metatarsal |  |
| C7 | Spinous process C7 |  |
| T10 | Spinous Process T10 |  |
