# Supplementary material for: Exploring lumbar and lower limb kinematics and kinetics for evidence that lifting technique is associated with LBP
Source: PLoS One. 2021 Jul 21;16(7):e0254241. doi: 10.1371/journal.pone.0254241 (PMC8294511; doi:10.1371/journal.pone.0254241)
Supplement: S2 Table — (DOCX) [file pone.0254241.s002.docx]

**S3 Table. Description of angular kinematics.**

|  | Images | Explanation of angle calculation |
| --- | --- | --- |
| KINEMATICS |  |  |
| Intra-lumbar flexion | 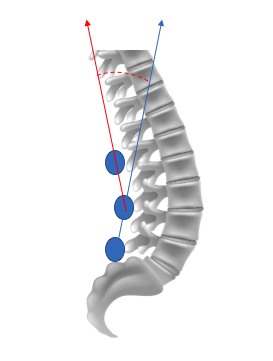 | Markers on L1, L3, L5 spinous process and also 5cm lateral to L2 and L4 spinous process bilaterally.  The current positioning indicates approximately -20°(red arrow). Negative values indicate more intra-lumbar extension (towards lordosis) and positive values indicate more intra-lumbar flexion (towards kyphosis). Vertical alignment of the upper lumbar and lower lumbar segments would equate to 0° of flexion. |
| Lumbo-pelvic flexion | 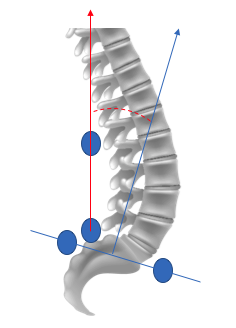 | Lumbar markers on L1 and L5 spinous process. Pelvis position was re-constructed from markers on ASIS and PSIS bilaterally. The arrowed blue line is orthogonal to the position of the ASIS and PSIS.  The current positioning indicates approximately -10° (red arrow). Negative values indicate lumbo-pelvic extension and positive values indicate lumbo-pelvic flexion. |
| Intra-lumbar lateral flexion | 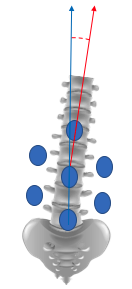 | Markers on L1, L3, L5 spinous process and also 5cm lateral to L2 and L4 spinous process bilaterally.  The current positioning indicates approximately 10° of right intra-lumbar lateral flexion. Vertical alignment of the upper and lower lumbar segment in the coronal plane would equate to 0° of lateral flexion. |
| Lumbo-pelvic lateral flexion | 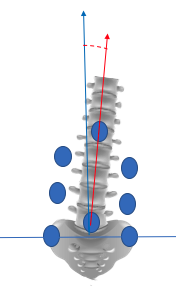 | Lumbar markers on L1 and L5 spinous process and also 5cm lateral to L2 and L4 spinous process bilaterally. Pelvis position was re-constructed from markers on ASIS and PSIS bilaterally.  The image shows approximately 10° of right lumbo-pelvic lateral flexion relative to the pelvic position. |
| Intra-lumbar rotation | 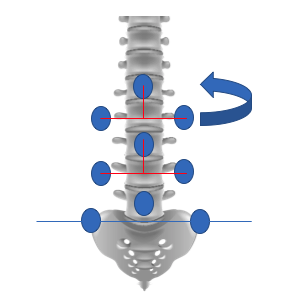 | Markers on L1, L3, L5 spinous process and also 5cm lateral to L2 and L4 spinous process bilaterally.  Alignment of the upper lumbar markers relative to the lower lumbar markers would equate to 0° intra-lumbar rotation. |
| Lumbo-pelvic rotation | 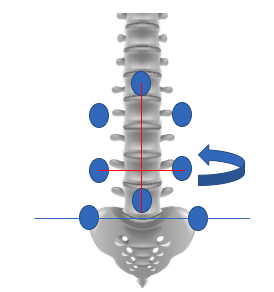 | Lumbar markers on L1 and L5 spinous process and also 5cm lateral to L2 and L4 spinous process bilaterally. Pelvis position was re-constructed from markers on ASIS and PSIS bilaterally.  Alignment of the lumbar markers relative to the pelvis in the would equate to 0° lumbo-pelvic rotation. |
| 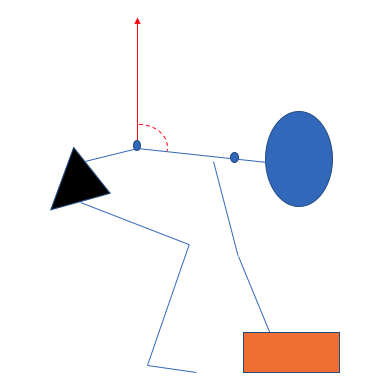Thorax inclination |  | Spinal markers on the C7 and T10 spinous process.  The image shows approximately 95° of thoracic inclination relative to the vertical. |
| Pelvic inclination  at box lift off | 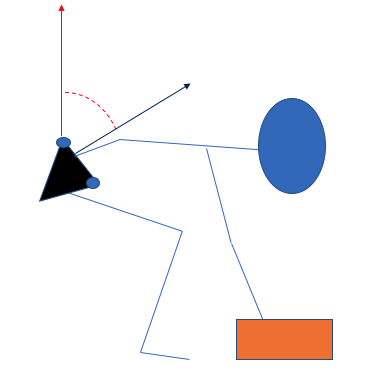 | The pelvis was re-constructed from markers on the ASIS and PSIS bilaterally. The arrowed blue line is orthogonal to the position of the ASIS and PSIS.  The image shows approximately 45° of pelvic inclination relative to the vertical. |
| Hip flexion | 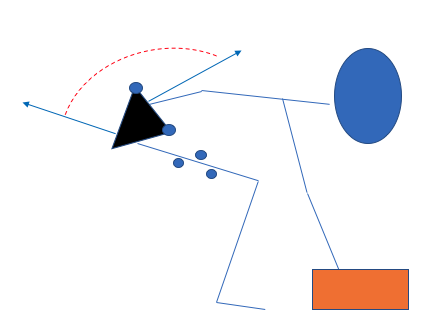 | The pelvis was re-constructed from markers on the ASIS and PSIS bilaterally. A three-marker rigid cluster was positioned on the lateral thigh. Hip position was equated by thigh cluster relative to the pelvis.  The image shows approximately 105° of hip flexion which was pelvis position relative to the thigh position. |
| Knee flexion | 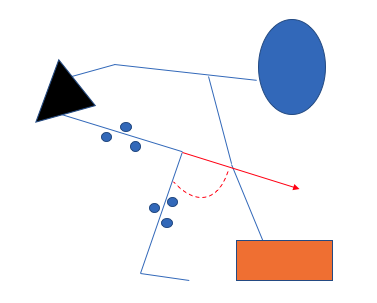 | A three-marker rigid cluster was positioned on the lateral thigh and anterior shin.  The image shows approximately 90° of knee flexion which was thigh position relative to the shin position. |
| Ankle dorsiflexion | **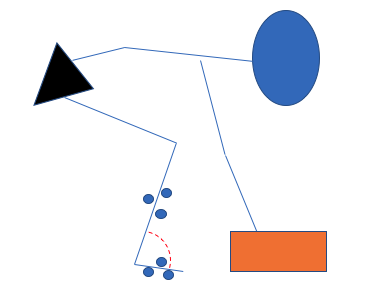** | A three-marker rigid cluster was positioned on the anterior shin and foot position was re-constructed from markers on the distal head of the 1^st^ and 5^th^ metatarsals and a calcaneal marker.  The image shows approximately 20° of dorsiflexion which is foot relative to tibia subtracted from 90° (neutral). |
| Heel lift from the floor (mm) | **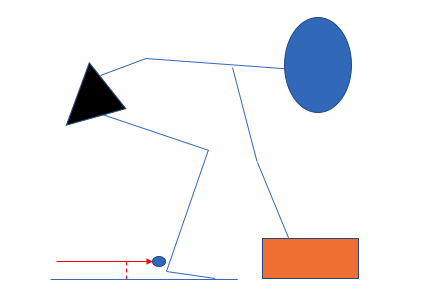** | A marker was positioned on the subtalar joint line and the distance from that marker to the floor was calculated. |
