# Supplementary material for: Exploring lumbar and lower limb kinematics and kinetics for evidence that lifting technique is associated with LBP
Source: PLoS One. 2021 Jul 21;16(7):e0254241. doi: 10.1371/journal.pone.0254241 (PMC8294511; doi:10.1371/journal.pone.0254241)
Supplement: S3 Table — (DOCX) [file pone.0254241.s003.docx]

**S5 Table: Kinematic and kinetic between group comparisons during lowering phase.**

|  | |  | | Group values (95%CI)  (unadjusted) | | |  | Difference  (unadjusted) | | Difference  (adjusted*) |
| --- | --- | --- | --- | --- | --- | --- | --- | --- | --- | --- |
| SPATIAL  KINEMATICS |  | |  | | | |  |  | |  |
|  |  | | LBP | | | | noLBP |  | |  |
| Peak  intra-lumbar  flexion‡ | **Lift 1**  **Lift 95** | | **15.1°**  **(13.0 to 17.1)** | | | | **20.1°**  **(18.0 to 22.2)** | **-5.0°**  **(-7.9 to -2.1)**  **P=0.001**  **-3.2°**  **(-5.5 to -0.3)**  **P=0.025** | | **-4.7°**  **(-8.0 to -1.4)**  **p=0.005**  **-3.4°**  **(-6.7 to -0.0)**  **P=0.049** |
|  |  |  | **19.6°**  **(17.2 to 21.9)** | | | | **22.8°**  **(21.1 to 24.4)** |  |  |  |
| Peak lumbo-pelvic flexion | Lift 1  Lift 95 | | 16.4°  (13.7 to 19.1) | | | | 16.6°  (14.2 to 19.0) | -0.1°  (-3.7 to 3.4)  P=0.935  1.5°  (-3.8 to 6.8)  P=0.585 | | 0.4°  (-3.4 to 4.1)  p=0.861  2.1°  (-3.1 to 7.4)  P=0.419 |
|  |  |  | 17.2°  (14.8 to 19.6) | | | | 15.7°  (11.3 to 20.2) |  |  |  |
| Peak intra-lumbar lateral flexion | Lift 1  Lift 95 | | 5.8°  (5.0 to 6.5) | | 5.2°  (4.3 to 6.0) | | | 0.6°  (-0.6 to 1.7)  P=0.337  0.6°  (-0.6 to 1.8)  P=0.315 | | 0.3°  (-0.9 to 1.6)  p=0.610  0.5°  (-0.9 to 2.0)  P=0.457 |
|  |  |  | 5.9°  (5.0 to 6.9) | | 5.3°  (4.5 to 6.0) | | |  |  |  |
| Peak lumbo-pelvic lateral flexion†‡ | Lift 1  Lift 95 | | 3.4°  (2.9 to 3.8) | | 3.4°  (2.8 to 4.0) | | | 0.0°  (-0.8 to 0.7)  P=0.932  -0.4°  (-1.2 to 0.4)  P=0.360 | | 0.0°  (-0.9 to 0.8)  p=0.944  -0.5°  (-1.7 to 0.7)  P=0.389 |
|  |  |  | 3.5°  (3.1 to 3.9) | | 3.9°  (3.2 to 4.6) | | |  |  |  |
| Peak intra-lumbar rotation‡ | Lift 1  Lift 95 | | 2.7°  (2.3 to 3.2) | | 2.6°  (2.3 to 2.9) | | | 0.1°  (-0.4 to 0.7)  P=0.648  0.2°  (-0.5 to 0.9)  P=0.543 | | 0.0°  (-0.6 to 0.7)  p=0.956  0.2°  (-0.6 to 1.0)  P=0.685 |
|  |  |  | 2.9°  (2.4 to 3.4) | | 2.7°  (2.4 to 3.1) | | |  |  |  |
| Peak lumbo-pelvic rotation | Lift 1  Lift 95 | | 3.1°  (2.6 to 3.7) | | 3.0°  (2.5 to 3.5) | | | 0.1°  (-0.6 to 0.8)  P=0.765  0.3°  (-0.4 to 1.0)  P=0.429 | | 0.2°  (-0.8 to 1.1)  p=0.722  0.5°  (-0.4 to 1.3)  P=0.314 |
|  |  |  | 3.3°  (2.7 to 3.9) | | 3.0°  (2.6 to 3.5) | | |  |  |  |
| Peak thoracic inclination†‡ | **Lift 1**  **Lift 95** | | **84.1°**  **(79.5 to 88.6)** | | | | **96.7°**  **(90.3 to 103.1)** | **-12.6°**  **(-20.9 to -4.4)**  **p=0.003**  **-4.0°**  **(-9.9 to 1.9)**  **p=0.184** | | **-14.4°**  **(-24.6 to -4.3)**  **p=0.005**  **-7.3°**  **(-14.9 to -0.9)**  **p=0.026** |
|  |  |  | **102.5°**  **(98.6 to 106.3)** | | | | **106.5°**  **(102.1 to 110.8)** |  |  |  |
| Pelvic inclination  at box lift off† | **Lift 1**  Lift 95 | | **38.5°**  **(33.3 to 43.8)** | | | | **51.0°**  **(44.7 to 57.3)** | **-12.5°**  **(-20.7 to -4.2)**  **p=0.003**  -4.0°  (-11.8 to 3.7)  p=0.307 | | **-15.8°**  **(-25.8 to -5.8)**  **p=0.002**  -6.7°  (-14.8 to 1.2)  p=0.097 |
|  |  |  | 50.0°  (45.0 to 55.0) | | | | 54.1°  (48.3 to 59.8) |  |  |  |
| Peak hip flexion | Lift 1  Lift 95 | | 107.2°  (104.6 to 109.8) | | | | 107.0°  (104.1 to 110.0) | 0.2°  (-3.6 to 3.9)  p= 0.926  -2.5°  (-8.4 to 3.4)  p= 0.407 | 1.2°  (-3.2 to 5.6)  p= 0.596  -1.5°  (-7.5 to 4.6)  p= 0.638 | |
|  |  |  | 107.0°  (104.3 to 109.6) | | | | 109.5°  (104.3 to 114.7) |  |  |  |
| Peak knee flexion† | **Lift 1**  Lift 95 | | **111.0°**  **(101.3 to 120.7.6)** | | | **88.3°**  **(75.7 to 101.0)** | | **22.6°**  **(6.6 to 38.7)**  **p=0.006**  6.8°  (-8.7 to 22.2)  p=0.392 | | **25.4°**  **(6.8 to 44.0)**  **p=0.007**  10.5°  (-6.6 to 27.7)  p=0.228 |
|  |  |  | 91.0°  (80.6 to 101.4) | | | 84.2°  (71.0 to 97.5) | |  |  |  |
| Peak ankle dorsiflexion† | **Lift 1**  Lift 95 | | **33.3°**  **(30.6 to 35.9)** | | | **24.4°**  **(20.4 to 28.4)** | | **8.8°**  **(3.9 to 13.8)**  **P<0.001**  2.8°  (-2.5 to 8.1)  p=0.302 | | **9.2°**  **(4.0 to 14.4)**  **p=0.001**  3.9°  (-1.2 to 9.1)  p=0.134 |
|  |  |  | 25.0°  (23.3 to 30.1) | | | 22.2°  (21.6 to 28.5) | |  |  |  |
| Peak heel lift (mm) | **Lift 1**  Lift 95 | | **59.8**  **(50.5 to 69.2)** | | | **44.2**  **(41.0 to 47.5)** | | **15.6**  **(6.2 to 25.1)**  **p= 0.001**  8.6  (-1.7 to 18.9)  p= 0.101 | | **14.6**  **(2.2 to 27.1)**  **p=0.021**  6.1  (-5.5 to 17.7)  p=0.307 |
|  |  |  | 56.8  (47.4 to 66.1) | | | 48.1  (44.5 to 51.8) | |  |  |  |
| TEMPORAL  KINEMATICS |  | |  | | |  | |  | |  |
| Peak lumbar (L1-L5) segment velocity relative to the vertical (deg/s)‡ | **Lift 1**  **Lift 95** | | **86.0 (78.6 to 93.3)** | | | **99.7 (91.3 to 108.0)** | | **-13.7 (-25.0 to -2.4)**  **P=0.018**  **-11.9 (-21.6 to -2.0)**  **p=0.017** | | **-18.3 (-29.5 to -7.0)**  **P=0.001**  **-13.1 (-25.2 to -1.1)**  **p=0.033** |
|  |  |  | **102.5 (96.2 to 108.7)** | | | **114.4 (106.9 to 121.9)** | |  |  |  |
| Average bending lumbar (L1-L5) segment velocity relative to the vertical (deg/s)‡ | **Lift 1**  Lift 95 | | **39.6 (34.6 to 44.6)** | | | **52.1 (46.6 to 57.6)** | | **-12.5 (-20.3 to -4.7)**  **P=0.002**  **-8.5 (-16.6 to -0.3)**  **p=0.041** | | **-14.9 (-22.9 to -6.8)**  **P<0.001**  -8.8 (-18.3 to 0.6)  p=0.067 |
|  |  |  | 49.3 (44.5 to 54.2) | | | 57.8 (51.6 to 64.1) | |  |  |  |
| Average lifting lumbar (L1-L5) segment velocity relative to the vertical (deg/s)†‡ | Lift 1  Lift 95 | | -27.8 (-32.8 to -22.8) | | | -30.9 (-35.7 to -26.1) | | 3.0 (-4.2 to 10.3)  P=0.411  -5.9 (-16.3 to 4.4)  p=0.261 | | 3.0 (-5.9 to 12.0)  P=0.503  -2.8 (-13.2 to 7.6)  p=0.599 |
|  |  |  | -26.4 (-34.2 to -18.6) | | | -20.4 (-27.7 to -13.2) | |  |  |  |
| Peak Thorax velocity (C7-T10 segment inclination deg/s)  ‡ | **Lift 1**  Lift 95 | | **105.0 (96.6 to 113.4)** | | | **119.7 (109.9 to 129.4)** | | **-14.7 (-28.3 to -1.1)**  **P=0.033**  -8.9 (-23.6 to 5.7)  p=0.232 | | **-21.8 (-35.7 to -8.0)**  **P=0.002**  -10.9 (-28.8 to 7.1)  p=0.235 |
|  |  |  | 129.4 (120.8 to 138.0) | | | 138.3 (126.3 to 150.4) | |  |  |  |
| Average Thorax velocity (C7-T10) segment inclination (bend with no box deg/s) | **Lift 1**  Lift 95 | | **50.7 (45.7 to 55.7)** | | | **63.2 (57.2 to 69.2)** | | **-12.5 (-20.8 to -4.9)**  **P=0.003**  -7.1 (-17.2 to 2.9)  p=0.162 | | **-16.0 (-24.9 to -7.4)**  **P<0.001**  -7.9 (-19.4 to 3.6)  P=0.179 |
|  |  |  | 63.6 (58.0 to 69.2) | | | 70.7 (62.6 to 78.8) | |  |  |  |
| Average Thorax velocity (C7-T10 segment inclination (lift with box deg/s) †‡ | Lift 1  Lift 95 | | -37.7 (-43.4 to -32.0) | | | -38.8 (-44.0 to -32.0) | | 1.1 (-7.0 to 9.1)  P=0.795  -10.4 (-22.4 to 1.7)  p=0.091 | | 0.5 (-9.3 to 10.4)  P=0.913  -5.4 (-17.2 to 6.3)  p=0.365 |
|  |  |  | -37.1 (-45.8 to -28.4) | | | -26.7 (-35.3 to -18.2) | |  |  |  |
| KINETICS |  | |  | | |  | |  | |  |
| Peak lumbar power (Normalised to body mass W/kg)  ‡ | Lift 1  Lift 95 | | 0.8 (0.7 to 0.9) | | | 0.8 (0.7 to 0.9) | | -0.0 (-0.2 to 0.2)  P=0.999  -0.1 (-0.2 to 0.0)  p=0.141 | | 0.0 (-0.1 to 0.2)  P=0.913  -0.0 (-0.1 to 0.1)  p=0.758 |
|  |  |  | 0.9 (0.8 to 1.0) | | | 1.0 (0.9 to 1.1) | |  |  |  |
| Average lumbar power (Normalised to body mass W/kg) (bend with no box)  ‡ | Lift 1  Lift 95 | | 0.4 (0.3 to 0.4) | | | 0.4 (0.3 to 0.4) | | -0.0 (-0.1 to 0.0)  P=0.464  -0.0 (-0.1 to 0.0)  p=0.206 | | -0.0 (-0.1 to 0.1)  P=0.634  -0.0 (-0.1 to 0.0)  P=0.479 |
|  |  |  | 0.4 (0.3 to 0.4) | | | 0.4 (0.4 to 0.5) | |  |  |  |
| Average lumbar power (Normalised to body mass W/kg) (lift with box)  ‡ | Lift 1  Lift 95 | | -0.3 (-0.3 to -0.2) | | | -0.3 (-0.3 to -0.2) | | -0.0 (-0.1 to 0.0)  P=0.589  -0.0 (-0.1 to -0.0)  p=0.257 | | -0.0 (-0.1 to 0.1)  P=0.895  -0.0 (-0.1 to 0.0)  P=0.209 |
|  |  |  | -0.3 (-0. to -0.2) | | | -0.2 (-0.3 to -0.1) | |  |  |  |
| Peak hip power (Normalised to body mass W/kg)  † | Lift 1  Lift 95 | | 1.4 (1.1 to 1.6) | | | 1.4 (1.3 to 1.6) | | -0.1 (-0.4 to 0.2)  P=0.539  0.4 (-0.1 to 0.9)  p=0.126 | | -0.1 (-0.4 to 0.3)  P=0.668  0.2 (-0.1 to 0.6)  p=0.221 |
|  |  |  | 1.6 (1.2 to 2.0) | | | 1.3 (1.0 to 1.5) | |  |  |  |
| Peak knee power (Normalised to body mass W/kg)  ‡ | **Lift 1**  **Lift 95** | | **1.1 (0.9 to 1.2)** | | | **0.7 (0.5 to 0.9)** | | **0.3 (0.1 to 0.6)**  **P=0.004**  **0.3 (0.0 to 0.5)**  **p=0.045** | | **0.2 (0.0 to 0.5)**  **P=0.023**  **0.3 (0.0 to 0.7)**  **p=0.028** |
|  |  |  | **0.8 (0.6 to 1.0)** | | | **0.6 (0.4 to 0.7)** | |  |  |  |
| Peak lumbar moment (Normalised to body mass) (NM/kg)‡ | Lift 1  Lift 95 | | 2.4 (2.3 to 2.5) | | | 2.4 (2.2 to 2.5) | | 0.0 (-0.1 to 0.2)  P=0.920  -0.1 (-0.3 to 0.2)  p=0.583 | | 0.0 (-0.2 to 0.2)  P=0.996  0.0 (-0.3 to 0.2)  p=0.690 |
|  |  |  | 2.7 (2.6 to 2.9) | | | 2.8 (2.6 to 3.0) | |  |  |  |
| Peak lumbar external anterior shear force (Normalised to body mass) (N/kg)‡ | **Lift 1**  Lift 95 | | **4.5 (4.2 to 4.8)** | | | **4.9 (4.6 to 5.2)** | | **-0.4 (-0.8 to 0.0)**  **P=0.032**  -0.3 (-0.8 to 0.1)  p=0.106 | | **-0.7 (-1.2 to -0.1)**  **P=0.010**  -0.3 (-0.8 to 0.1)  p=0.103 |
|  |  |  | 5.8 (5.4 to 6.1) | | | 6.1 (5.8 to 6.1) | |  |  |  |
| Peak lumbar lateral shear force (Normalised to body mass) (N/kg) †‡ | Lift 1  Lift 95 | | 0.5 (0.4 to 0.5) | | | 0.5 (0.4 to 0.6) | | 0.0 (-0.1 to 0.0)  P=0.320  -0.1 (-0.2 to 0.0)  p=0.054 | | -0.1 (-0.2 to 0.1)  P=0.239  -0.1 (-0.3 to 0.0)  p=0.175 |
|  |  |  | 0.6 (0.6 to 0.7) | | | 0.8 (0.7 to 0.9) | |  |  |  |
| Peak lumbar external compression force (Normalised to body mass) (N/kg)‡ | Lift 1  Lift 95 | | 4.2 (4.1 to 4.3) | | | 4.3 (3.9 to 4.7) | | -0.1 (-0.5 to 0.3)  P=0.681  -0.1 (-0.6 to 0.4)  p=0.697 | | 0.0 (-0.5 to 0.5)  P=0.917  -0.2 (-0.9 to 0.4)  p=0.472 |
|  |  |  | 4.8 (4.6 to 5.0) | | | 4.9 (4.5 to 5.3) | |  |  |  |

*Adjusted for age, sex, height, weight, an interaction between pain group and lift type, an interaction between box weight and lift type and lift number.

**†** Significant interaction between group and time.

**‡** Significant time effect.
