# Supplementary material for: Exploring lumbar and lower limb kinematics and kinetics for evidence that lifting technique is associated with LBP
Source: PLoS One. 2021 Jul 21;16(7):e0254241. doi: 10.1371/journal.pone.0254241 (PMC8294511; doi:10.1371/journal.pone.0254241)
Supplement: S4 Table — (DOCX) [file pone.0254241.s004.docx]

**S6 Table. Associations between pain ramp during the lowering phase and each kinematic or kinetic variable that was different between groups - Associations for the LBP group only.**

|  | Unadjusted  Coefficient  (95% CI) | | p-value | Adjusted*  Coefficient  (95% CI) | p-value |
| --- | --- | --- | --- | --- | --- |
| *Lowering phase - Spatial kinematics* |  | |  |  |  |
| Peak intra-lumbar flexion | 0.006 (-0.013 to 0.026) | | 0.505 | 0.009 (-0.014 to 0.026) | 0.554 |
| Peak thorax inclination (C7-T10 segment inclination relative to the vertical) | 0.002 (-0.006 to 0.010) | | 0.617 | 0.002 (-0.006 to 0.010) | 0.633 |
| Peak knee flexion | -0.002 (-0.008 to 0.004) | | 0.509 | -0.002 (-0.008 to 0.004) | 0.518 |
| Peak ankle dorsiflexion | -0.013 (-0.027 to 0.002) | | 0.085 | -0.012 (-0.027 to 0.019) | 0.089 |
| Peak heel lift | 0.004 (-0.005 to 0.012) | | 0.404 | 0.004 (-0.005 to 0.012) | 0.380 |
| Pelvic inclination at box lift off | 0.002 (-0.008 to 0.013) | | 0.645 | 0.002 (0.008 to 0.013) | 0.669 |
| *Lowering phase - Temporal kinematics* | |  |  |  |  |
| Average lumbar velocity during unloaded return phase (L1-L5 segment inclination relative to the vertical) | -0.002 (-0.010 to 0.006) | | 0.595 | -0.010 (-0.004 to 0.006) | 0.628 |
| Average thorax velocity during unloaded return phase (C7-T10 segment inclination relative to the vertical) | -0.001 (-0.007 to 0.005) | | 0.676 | -0.001 (-0.007 to 0.005) | 0.720 |
| *Lowering phase - kinetics* |  | |  |  |  |
| Peak knee power | 0.031 (-0.171 to 0.233) | | 0.766 | 0.030 (-0.174 to 0.234) | 0.771 |

* Adjusted for age and sex
