# Supplementary material for: Exploring lumbar and lower limb kinematics and kinetics for evidence that lifting technique is associated with LBP
Source: PLoS One. 2021 Jul 21;16(7):e0254241. doi: 10.1371/journal.pone.0254241 (PMC8294511; doi:10.1371/journal.pone.0254241)
Supplement: S5 Table — For this sensitivity analysis, those participants (6, 11 and 18) who did not experience pain increase with lifting were removed. (DOCX) [file pone.0254241.s005.docx]

**S7 Table. In those workers with a history of low back pain (LBP group), associations between pain ramp during the lifting and lowering phases and each kinematic or kinetic variable that was different between groups. For this sensitivity analysis, those participants (6, 11 and 18) who did not experience pain increase with lifting were removed.**

|  | Unadjusted  Coefficient  (95% CI) | | p-value | Adjusted*  Coefficient  (95% CI) | p-value |
| --- | --- | --- | --- | --- | --- |
| *Lifting Phase - Spatial kinematics* |  | |  |  |  |
| Peak intra-lumbar flexion | 0.012 (-0.011 to 0.034) | | 0.303 | 0.011 (-0.011 to 0.034) | 0.330 |
| Peak thorax inclination (C7-T10 segment inclination relative to the vertical) | 0.005 (-0.005 to 0.015) | | 0.324 | 0.005 (-0.005 to 0.016) | 0.296 |
| Peak knee flexion | -0.004 (-0.010 to 0.002) | | 0.221 | -0.004 (-0.010 to 0.002) | 0.213 |
| Peak ankle dorsiflexion | -0.015 (-0.037 to 0.007) | | 0.189 | -0.014 (-0.035 to 0.008) | 0.211 |
| Peak heel lift | -0.002 (-0.008 to 0.003) | | 0.493 | -0.002 (-0.008 to 0.003) | 0.421 |
| Pelvic inclination at box lift off | 0.010 (-0.002 to 0.021) | | 0.100 | 0.010 (0.010 to 0.006) | 0.096 |
| *Lifting phase - Temporal kinematics* | |  |  |  |  |
| Peak lumbar velocity (L1-L5 segment inclination relative to the vertical) | 0.003 (-0.003 to 0.008) | | 0.334 | 0.003 (-0.003 to 0.008) | 0.340 |
| Average lumbar velocity during unloaded bending phase (L1-L5 segment inclination relative to the vertical) | 0.005 (-0.002 to 0.012) | | 0.130 | 0.005 (-0.002 to 0.012) | 0.137 |
| Peak thorax velocity (C7-T10 segment inclination relative to the vertical) | 0.001 (-0.003 to 0.006) | | 0.466 | 0.001 (-0.003 to 0.005) | 0.489 |
| Average thorax velocity during unloaded bending phase (C7-T10 segment inclination relative to the vertical) | 0.004 (-0.002 to 0.009) | | 0.171 | 0.004 (-0.002 to 0.009) | 0.178 |
| *Lifting phase - kinetics* |  | |  |  |  |
| Peak knee power | -0.006 (-0.135 to 0.123) | | 0.930 | -0.002 (-0.126 to 0.122) | 0.972 |
| Peak external anterior shear force | 0.119 (-0.091 to 0.330) | | 0.266 | 0.122 (-0.088 to 0.332) | 0.256 |
| *Lowering phase - Spatial kinematics* |  | |  |  |  |
| Peak intra-lumbar flexion | 0.009 (-0.011 to 0.030) | | 0.382 | 0.009 (-0.013 to 0.030) | 0.426 |
| Peak thorax inclination (C7-T10 segment inclination relative to the vertical) | 0.002 (-0.007 to 0.011) | | 0.690 | 0.002 (-0.007 to 0.011) | 0.678 |
| Peak knee flexion | -0.003 (-0.009 to 0.004) | | 0.442 | -0.002 (-0.008 to 0.004) | 0.445 |
| Peak ankle dorsiflexion | -0.013 (-0.030 to 0.003) | | 0.122 | -0.012 (-0.029 to 0.004) | 0.146 |
| Peak heel lift | -0.002 (-0.013 to 0.010) | | 0.771 | -0.002 (-0.014 to 0.009) | 0.676 |
| Pelvic inclination at box lift off | 0.004 (-0.006 to 0.013) | | 0.447 | 0.003 (-0.006 to 0.013) | 0.470 |
| *Lowering phase - Temporal kinematics* | |  |  |  |  |
| Average lumbar velocity during unloaded return phase (L1-L5 segment inclination relative to the vertical) | -0.004 (-0.013 to 0.004) | | 0.299 | -0.004 (-0.013 to 0.004) | 0.308 |
| Average thorax velocity during unloaded return phase (C7-T10 segment inclination relative to the vertical) | -0.002 (-0.009 to 0.004) | | 0.459 | -0.002 (-0.009 to 0.004) | 0.486 |
| *Lowering phase - kinetics* |  | |  |  |  |
| Peak knee power | -0.004 (-0.141 to 0.132) | | 0.951 | -0.001 (-0.136 to 0.134) | 0.992 |

* Adjusted for age and sex
