# Supplementary material for: Exploring lumbar and lower limb kinematics and kinetics for evidence that lifting technique is associated with LBP
Source: PLoS One. 2021 Jul 21;16(7):e0254241. doi: 10.1371/journal.pone.0254241 (PMC8294511; doi:10.1371/journal.pone.0254241)
Supplement: S1 File — (DOCX) [file pone.0254241.s006.docx]

**S1 File. Lift testing procedures.**

A marker set was placed on relevant bony landmarks. Subsequently, a static calibration trial was collected.

The box lifting task was adapted from Lariviere et al (1). The dimensions of the box were 400mm (L) x 200mm (W) x 100mm (H) with hand holes (top of the opening at 70mm from box base). Lifts 1-25 were performed with the empty box (200 grams) then lifts 26-100 were completed with a box equivalent to 10% of body mass. The lifting task was continuous and the following 5 lift types were completed in sequence 20 times:

1. Symmetrical - floor height (origin) to hip height and return to floor (destination).
2. Asymmetrical - floor height in front (origin) to floor height 45 degrees to the left (destination).
3. Asymmetrical - floor height 45 degrees left (origin) to floor height in front (destination).
4. Asymmetrical - floor height in front (origin) to floor height 45 degrees to the right (destination).
5. Asymmetrical - floor height 45 degrees right (origin) to floor height in front (destination).

Participants received only verbal instructions, with no demonstration of lifting technique or how the task should be performed. They were instructed to complete the task and lift in a way that they would normally.

1. Lariviere C, Gagnon D, Loisel P. A biomechanical comparison of lifting techniques between subjects with and without chronic low back pain during freestyle lifting and lowering tasks. Clinical biomechanics (Bristol, Avon). 2002;17(2):89-98.
