# Supplementary material for: Exploring lumbar and lower limb kinematics and kinetics for evidence that lifting technique is associated with LBP
Source: PLoS One. 2021 Jul 21;16(7):e0254241. doi: 10.1371/journal.pone.0254241 (PMC8294511; doi:10.1371/journal.pone.0254241)
Supplement: S2 File — (DOCX) [file pone.0254241.s007.docx]

**S4 File. Biomechanical modelling.**

Vicon Nexus motion analysis software (Oxford metrics, Oxford, U.K.) was used for the processing of data. Marker trajectories were checked for ‘breaks’ or missing information that can occur as a result of marker occlusion. Standard procedures were used to interpolate these missing data, which were small and infrequent.

A mathematical model was created in order to perform all biomechanics calculations. The upper lumbar region was defined from; a Y axis from the L3 to the L1 marker, the cross product of this axis and a line connecting the two markers bilateral of the L2/L3 junction was used to create the Z axis, and finally the cross product of the Y and Z axes was used to create the X axis. The lower lumbar region was calculated as; Y axis was defined from the L5 to the L3 marker, the cross product of this axis and a line connecting the two markers bilateral of the L4/L5 junction was used to create the Z axis, and finally the cross product of the Y and Z axes was used to create the X axis. Intra-lumbar spine flexion was calculated as the relative angle between the upper and lower lumbar regions using a Z-X-Y Eular angle decomposition.
